# Crystal structure of (*S*)-*sec*-butyl­ammonium l-tartrate monohydrate

**DOI:** 10.1107/S2056989017005448

**Published:** 2017-04-18

**Authors:** Ernlie A. Publicover, Jennifer Kolwich, Darcie L. Stack, Alyssa J. Doué, Kai E. O. Ylijoki

**Affiliations:** a Department of Chemistry, Saint Mary’s University, 923 Robie St., Halifax, NS, B3H 3C3, Canada

**Keywords:** crystal structure, *sec*-butyl­amine, l-tartaric acid, chiral resolution, monohydrate, hydrogen bonding

## Abstract

The title hydrated mol­ecular salt, C_4_H_12_N^+^·C_4_H_5_O_6_^−^·H_2_O, was prepared by deprotonation of enanti­opure l-tartaric acid with racemic *sec*-butyl­amine in water. Only one enanti­omer was observed crystallographically, resulting from the combination of (*S*)-*sec*-butyl­amine with l-tartaric acid. The *sec*-butyl­ammonium moiety is disordered over two conformations related by rotation around the CH–CH_2_ bond; the refined occupancy ratio is 0.68 (1):0.32 (1). In the crystal, mol­ecules are linked through a network of O—H⋯O and N—H⋯O hydrogen-bonding inter­actions, between the ammonium H atoms, the tartrate hy­droxy H atoms, and the inter­stitial water, forming a three-dimensional supra­molecular structure.

## Abstract 2

The title hydrated mol­ecular salt was prepared by deprotonation of enanti­opure l-tartaric acid with racemic *sec*-butyl­amine in water. Only one enanti­omer was observed crystallographically, resulting from the combination of (*S*)-*sec*-butyl­amine with l-tartaric acid.

## Chemical context   

Given that the two enanti­omers of chiral compounds can display significantly different reactivity in the presence of other chiral compounds (*e.g.*, enzymatic reactions), the separation of racemic mixtures is an important process in chemical synthesis. Since enanti­omers have identical physical properties, they cannot be separated by standard physical means such as distillation, crystallization, or chromatography. One common method to overcome this issue is to convert the racemic compound into a mixture of diastereomers through reaction with an enanti­opure component (Fogassy *et al.*, 2006 ▸). This method has been used for the resolution of amine enanti­omers by protonation with chiral tartaric acid to produce diastereomeric salts. Examples include resolution of α-phenyl­ethyl­amine (Ault 1965 ▸; Kokila *et al.*, 2002 ▸), *N*-methyl­amphetamines (Kmecz *et al.*, 2004 ▸), 2-(benzyl­amino)-4-oxo-4-phenyl­butano­ate (Berkeš *et al.*, 2003 ▸), 3-amino­butanol (Yatcherla *et al.*, 2015 ▸), aminona­phthols (Periasamy *et al.*, 2009 ▸), and serotonin and dopamine antagonists (Campiani *et al.*, 2002 ▸).
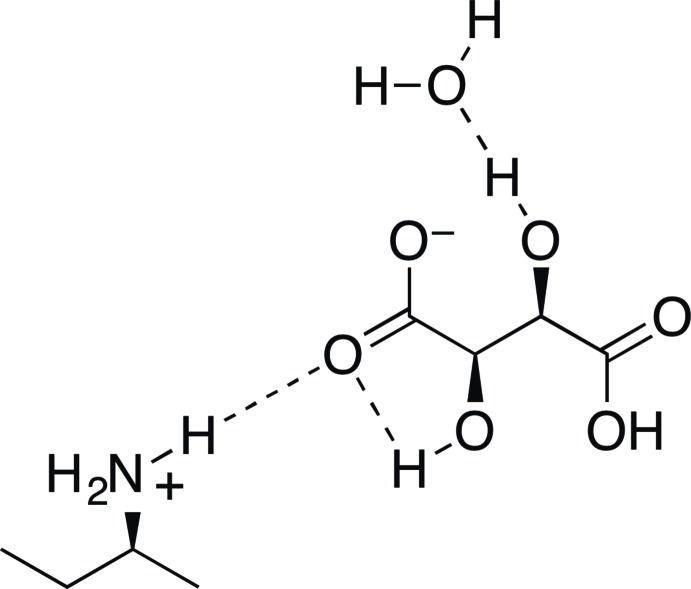


## Structural commentary   

**Figure 1:**
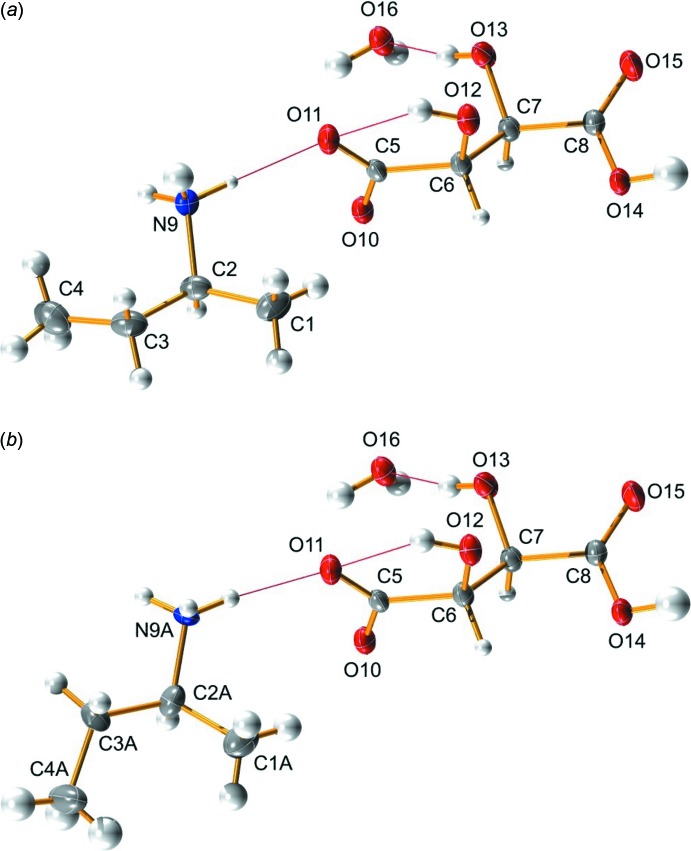
The mol­ecular structure of the title hydrated mol­ecular salt, showing (*a*) the major and (*b*) the minor components of the disordered *sec*-butyl­ammonium moiety. Displacement ellipsoids are drawn at the 50% probability level. Red lines indicate the hydrogen bonds present within the asymmetric unit (see Table 1 ▸).

The mol­ecular structure of the title hydrated mol­ecular salt is shown in Fig. 1 ▸. The salt crystallized as a single enanti­omer, consisting of an (*S*)-*sec*-butyl ammonium cation, the l-tartrate anion, and one mol­ecule of water in the asymmetric unit. The Flack parameter [–2.7 (8)] was not of use in determining the absolute configuration of the *sec*-butyl­amine in the crystal. The absolute configuration of the (*S*)-*sec*-butyl ammonium cation is therefore based on the known absolute configuration of the l-tartaric acid used during compound preparation. The final structure is disordered, with the *sec*-butyl ammonium moiety taking on two different rotamers about the C2–C3 axis [refined occupancy ratio is 0.68 (1):0.32 (1)]. The major component takes on a conformation where the C4 methyl group and N9 ammonium are in a *gauche* relationship (Fig. 1 ▸ *a*), while the minor component places the C4*A* methyl group anti­periplanar to the N9*A* ammonium (Fig. 1 ▸ *b*). The C—C bond lengths in the amine and tartrate units average 1.523 (11) Å [1.516 (22) Å for the minor component of the disorder] and 1.532 (5) Å, respectively. The C—N bonds of the two components of the disorder average 1.498 (17) Å. The tartrate C—OH bonds average 1.411 (4) Å, while the C—O bonds of the carboxyl moieties average 1.257 (4) Å for the one involved in hydrogen bonding with the amine, and 1.258 (4) Å for the other. An intra­molecular hydrogen bond [2.00 (3) Å] occurs with O12 acting as a hydrogen-bond donor to O11.

## Supra­molecular features   

**Figure 2:**
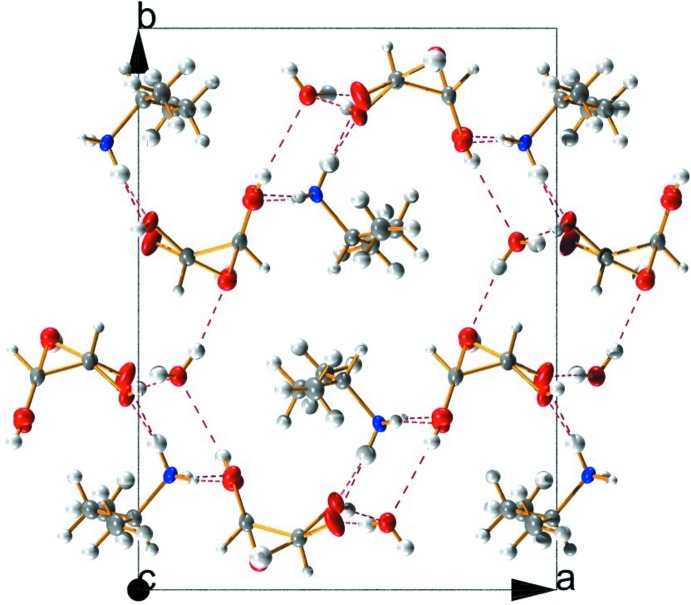
A view of the crystal packing of the title hydrated mol­ecular salt, viewed along the *c* axis (major component of the disorder only). Red dashed lines indicate the inter­molecular hydrogen-bonding network (see Table 1 ▸). Displacement ellipsoids are drawn at the 50% probability level.

The supra­molecular structure of the crystal consists of a network of inter­molecular O—H⋯O and N—H⋯O hydrogen bonds (Table 1 ▸, Fig. 2 ▸). Within the asymmetric unit, the N9—H9*A* atom of the *sec*-butyl ammonium cation acts as a hydrogen-bond donor to O11 of the tartrate anion [1.89 (2) Å], and the tartrate O13 donates a hydrogen bond to O16 of water [1.83 (3) Å]. The water in turn acts as a hydrogen-bond donor to O10 [2.01 (3) Å] and O15 [1.93 (4) Å] of two adjacent symmetry-related mol­ecules. Three additional hydrogen bonds are formed from N9, with N9—H9*B* donating to O12 of an adjacent mol­ecule [1.97 (3) Å], and N9—H9*C* donating to both O13 [2.16 (4) Å] and O15 [2.20 (4) Å] of a second adjacent mol­ecule. Finally, O14 donates a hydrogen bond to O10 of an additional symmetry-related mol­ecule [1.58 (5) Å]. A view of the crystal packing reveals the amine, tartrate, and water mol­ecules form columns when viewed down the *c* axis (Fig. 2 ▸).

## Database survey   

The Cambridge Structural Database (CSD, Version 5.37; Groom *et al.*, 2016 ▸) does not contain any other examples of simple secondary alkyl ammonium tartrate compounds. Two primary alkyl ammonium compounds have been reported: methyl­ammonium l-tartrate (XOJMOA; Callear *et al.*, 2008*a* ▸) and *n*-butyl ammonium tartrate monohydrate (XOJDIL; Callear *et al.*, 2008*b* ▸). Multiple stereoisomers of the phenyl­ethyl­ammonium tartrate salt have also been reported, *viz.* BUSHED (Mei *et al.*, 2010 ▸), JADTUD (Molins *et al.*, 1989 ▸), QAMYIN (Turkington *et al.*, 2005 ▸), along with the related napthylethyl ammonium tartrate (QAPTEG; Gül & Nelson, 1999 ▸).

## Synthesis and crystallization   

The title compound was prepared *via* a modification to a previously published procedure (Helmkamp & Johnson, 1983 ▸). Racemic *sec*-butyl­amine (23.7 g, 17.2 ml, 324.0 mmol) was added to 40 ml of water and stirred to ensure homogeneity. While stirring, l-tartaric acid (50.0 g, 333.1 mmol) was slowly added. The solution was covered and allowed to stand at ambient temperature. After 24 h, crystal formation was evident. The crystallization process was allowed to continue undisturbed for one week, at which point a crystal for diffraction analysis was selected directly from the reaction mixture without further purification or isolation. The crystals can be isolated by vacuum filtration to yield a white crystalline solid (33.5 g, 42%).

## Refinement   

Crystal data, data collection, and structure refinement details are summarized in Table 2 ▸. The H atoms on the N and O atoms were located in a difference-Fourier map and freely refined. The alkyl H atoms were included at geometrically idealized positions (C—H = 0.98–1.00 Å) and treated as riding with *U*_iso_(H) = 1.5*U*_eq_(C-meth­yl) and 1.2*U*_eq_(C) for other H atoms. The *sec*-butyl ammonium moiety displays a twofold disorder arising from two different rotamers being present that is best described as a 0.68 (1):0.32 (1) ratio of the two possible conformations. In the final cycles of refinement SAME restraints were applied to the two components of the disordered *sec*-butyl ammonium moiety and DFIX restraints were applied to the N—H bonds [N—H = 0.91 (2) Å] and the ammonium H⋯H distances [H⋯H = 1.50 (2) Å], to improve the refinement and geometry.

## Supplementary Material

Crystal structure: contains datablock(s) I, Global. DOI: 10.1107/S2056989017005448/su5364sup1.cif

Structure factors: contains datablock(s) I. DOI: 10.1107/S2056989017005448/su5364Isup2.hkl

Supporting information file. DOI: 10.1107/S2056989017005448/su5364Isup3.cml

CCDC reference: 1543331

Additional supporting information:  crystallographic information; 3D view; checkCIF report

## Figures and Tables

**Table 1:** Hydrogen-bond geometry (Å, °)

*D*—H⋯*A*	*D*—H	H⋯*A*	*D*⋯*A*	*D*—H⋯*A*
O12—H12⋯O11	0.90 (3)	2.00 (3)	2.602 (2)	123 (3)
O13—H13⋯O16	0.85 (3)	1.83 (3)	2.662 (2)	167 (3)
O14—H14⋯O10^i^	0.93 (4)	1.58 (5)	2.499 (2)	171 (5)
O16—H16*A*⋯O15^ii^	0.87 (4)	1.93 (4)	2.791 (2)	169 (4)
O16—H16*B*⋯O10^iii^	0.83 (4)	2.01 (3)	2.822 (2)	167 (3)
N9—H9*A*⋯O11	0.93 (2)	1.89 (2)	2.803 (9)	167 (4)
N9—H9*B*⋯O12^ii^	0.91 (2)	1.97 (3)	2.869 (11)	169 (4)
N9—H9*C*⋯O13^iv^	0.92 (2)	2.16 (4)	2.922 (13)	140 (5)
N9—H9*C*⋯O15^iv^	0.92 (2)	2.20 (4)	3.001 (12)	145 (5)
N9*A*—H9*AA*⋯O11	0.91 (3)	1.87 (4)	2.76 (2)	164 (8)
N9*A*—H9*AB*⋯O13^iv^	0.90 (3)	1.96 (6)	2.79 (3)	151 (9)
N9*A*—H9*AB*⋯O15^iv^	0.90 (3)	2.21 (8)	2.83 (3)	126 (6)
N9*A*—H9*AC*⋯O12^ii^	0.91 (3)	1.99 (5)	2.81 (3)	150 (7)

Symmetry codes: (i) 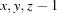; (ii) 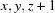; (iii) 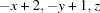; (iv) 
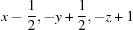
.

**Table 2:** Experimental details

Crystal data	
Chemical formula	C_4_H_12_N^+^·C_4_H_5_O_6_^−^·H_2_O
*M*_r_	241.24
Crystal system, space group	Orthorhombic, *P*2_1_2_1_2
Temperature (K)	125
*a*, *b*, *c* (Å)	11.0921 (10), 14.8876 (14), 7.2070 (7)
*V* (Å^3^)	1190.13 (19)
*Z*	4
Radiation type	Mo *K*α
μ (mm^−1^)	0.12
Crystal size (mm)	0.21 × 0.09 × 0.04

Data collection	
Diffractometer	Bruker APEXII CCD
Absorption correction	Multi-scan (*SADABS*; Bruker, 2008 ▸)
*T*_min_, *T*_max_	0.567, 0.746
No. of measured, independent and observed [*I* > 2σ(*I*)] reflections	9652, 2925, 2613
*R*_int_	0.067
(sin θ/λ)_max_ (Å^−1^)	0.680

Refinement	
*R*[*F* ^2^ > 2σ(*F* ^2^)], *wR*(*F* ^2^), *S*	0.040, 0.104, 1.03
No. of reflections	2925
No. of parameters	236
No. of restraints	20
H-atom treatment	H atoms treated by a mixture of independent and constrained refinement
Δρ_max_, Δρ_min_ (e Å^−3^)	0.30, −0.27

Computer programs: *APEX2* and *SAINT* (Bruker, 2008 ▸), *SHELXT* (Sheldrick, 2015*a* ▸), *SHELXL* (Sheldrick, 2015*b* ▸), *DIAMOND* (Crystal Impact, 2014 ▸) and *OLEX2* (Dolomanov *et al.*, 2009 ▸).

## supplementary crystallographic information

### Crystal data

**Table:**

C_4_H_12_N^+^·C_4_H_5_O_6_^−^·H_2_O	*D*_x_ = 1.346 Mg m^−^^3^
*M**_r_* = 241.24	Mo *K*α radiation, λ = 0.71073 Å
Orthorhombic, *P*2_1_2_1_2	Cell parameters from 5745 reflections
*a* = 11.0921 (10) Å	θ = 2.3–28.6°
*b* = 14.8876 (14) Å	µ = 0.12 mm^−^^1^
*c* = 7.2070 (7) Å	*T* = 125 K
*V* = 1190.13 (19) Å^3^	Needle, clear light colourless
*Z* = 4	0.21 × 0.09 × 0.04 mm
*F*(000) = 520	

### Data collection

**Table:**

Bruker APEXII CCD diffractometer	2613 reflections with *I* > 2σ(*I*)
Graphite monochromator	*R*_int_ = 0.067
φ and ω scans	θ_max_ = 28.9°, θ_min_ = 2.3°
Absorption correction: multi-scan (SADABS; Bruker, 2008)	*h* = −14→14
*T*_min_ = 0.567, *T*_max_ = 0.746	*k* = −19→20
9652 measured reflections	*l* = −9→9
2925 independent reflections	

### Refinement

**Table:**

Refinement on *F*^2^	Primary atom site location: dual
Least-squares matrix: full	Secondary atom site location: difference Fourier map
*R*[*F*^2^ > 2σ(*F*^2^)] = 0.040	Hydrogen site location: mixed
*wR*(*F*^2^) = 0.104	H atoms treated by a mixture of independent and constrained refinement
*S* = 1.03	*w* = 1/[σ^2^(*F*_o_^2^) + (0.0447*P*)^2^ + 0.1359*P*] where *P* = (*F*_o_^2^ + 2*F*_c_^2^)/3
2925 reflections	(Δ/σ)_max_ < 0.001
236 parameters	Δρ_max_ = 0.30 e Å^−^^3^
20 restraints	Δρ_min_ = −0.27 e Å^−^^3^

### Special details

**Table:**

Geometry. All esds (except the esd in the dihedral angle between two l.s. planes) are estimated using the full covariance matrix. The cell esds are taken into account individually in the estimation of esds in distances, angles and torsion angles; correlations between esds in cell parameters are only used when they are defined by crystal symmetry. An approximate (isotropic) treatment of cell esds is used for estimating esds involving l.s. planes.

### Fractional atomic coordinates and isotropic or equivalent isotropic displacement parameters (Å^2^)

**Table:**

	*x*	*y*	*z*	*U*_iso_*/*U*_eq_	Occ. (<1)
O10	0.78822 (15)	0.45123 (9)	0.6188 (2)	0.0219 (3)	
O11	0.72627 (15)	0.30780 (10)	0.6134 (2)	0.0250 (4)	
O12	0.72284 (14)	0.29621 (10)	0.2532 (2)	0.0225 (3)	
H12	0.702 (3)	0.264 (2)	0.353 (4)	0.047 (9)*	
O13	0.97215 (14)	0.34543 (11)	0.3063 (2)	0.0232 (4)	
H13	1.001 (3)	0.358 (2)	0.412 (4)	0.041 (9)*	
O14	0.78763 (14)	0.44450 (10)	−0.0347 (2)	0.0212 (3)	
H14	0.792 (4)	0.442 (3)	−0.163 (6)	0.092 (15)*	
O15	0.96848 (16)	0.37910 (14)	−0.0496 (3)	0.0382 (5)	
C5	0.75823 (18)	0.37845 (14)	0.5374 (3)	0.0177 (4)	
C6	0.75836 (18)	0.38090 (14)	0.3251 (3)	0.0181 (4)	
H6	0.698877	0.427117	0.283210	0.022*	
C7	0.88300 (18)	0.40657 (13)	0.2505 (3)	0.0184 (4)	
H7	0.904588	0.467567	0.298169	0.022*	
C8	0.88361 (19)	0.40887 (13)	0.0376 (3)	0.0189 (4)	
O16	1.09484 (15)	0.38045 (11)	0.6154 (2)	0.0239 (4)	
H16A	1.048 (3)	0.383 (3)	0.713 (6)	0.057 (11)*	
H16B	1.139 (3)	0.425 (2)	0.614 (5)	0.049 (10)*	
C1	0.4453 (5)	0.3844 (4)	0.7178 (9)	0.0343 (12)	0.683 (8)
H1A	0.409648	0.328062	0.673732	0.052*	0.683 (8)
H1B	0.513909	0.400409	0.638753	0.052*	0.683 (8)
H1C	0.384759	0.432249	0.712719	0.052*	0.683 (8)
C2	0.4888 (4)	0.3725 (4)	0.9196 (8)	0.0251 (11)	0.683 (8)
H2	0.532125	0.428349	0.957990	0.030*	0.683 (8)
C3	0.3852 (3)	0.3571 (3)	1.0525 (6)	0.0325 (11)	0.683 (8)
H3A	0.323796	0.404227	1.031552	0.039*	0.683 (8)
H3B	0.347576	0.298478	1.023235	0.039*	0.683 (8)
C4	0.4201 (4)	0.3577 (3)	1.2555 (6)	0.0386 (12)	0.683 (8)
H4A	0.469675	0.304846	1.282853	0.058*	0.683 (8)
H4B	0.347090	0.356292	1.332028	0.058*	0.683 (8)
H4C	0.466033	0.412306	1.282961	0.058*	0.683 (8)
N9	0.5762 (8)	0.2958 (8)	0.9253 (15)	0.0176 (14)	0.683 (8)
H9C	0.544 (7)	0.240 (3)	0.909 (7)	0.07 (2)*	0.683 (8)
H9A	0.634 (3)	0.305 (3)	0.834 (5)	0.015 (11)*	0.683 (8)
H9B	0.615 (4)	0.300 (3)	1.037 (4)	0.030 (12)*	0.683 (8)
C1A	0.4346 (13)	0.3806 (9)	0.8007 (19)	0.040 (3)	0.317 (8)
H1AA	0.387949	0.331055	0.747348	0.060*	0.317 (8)
H1AB	0.494591	0.401206	0.710339	0.060*	0.317 (8)
H1AC	0.380246	0.430337	0.831645	0.060*	0.317 (8)
C2A	0.4980 (13)	0.3487 (9)	0.9750 (18)	0.034 (3)	0.317 (8)
H2A	0.544271	0.400926	1.025413	0.041*	0.317 (8)
C3A	0.4163 (8)	0.3153 (6)	1.1293 (12)	0.033 (2)	0.317 (8)
H3AA	0.365209	0.266424	1.079528	0.039*	0.317 (8)
H3AB	0.467213	0.289296	1.228555	0.039*	0.317 (8)
C4A	0.3352 (11)	0.3859 (7)	1.2147 (17)	0.058 (4)	0.317 (8)
H4AA	0.281880	0.410607	1.118991	0.088*	0.317 (8)
H4AB	0.384600	0.434191	1.267114	0.088*	0.317 (8)
H4AC	0.286538	0.358557	1.313144	0.088*	0.317 (8)
N9A	0.588 (2)	0.2777 (18)	0.925 (4)	0.022 (4)	0.317 (8)
H9AA	0.645 (7)	0.290 (8)	0.837 (10)	0.027*	0.317 (8)
H9AB	0.535 (8)	0.236 (6)	0.885 (12)	0.027*	0.317 (8)
H9AC	0.623 (7)	0.263 (6)	1.034 (7)	0.027*	0.317 (8)

### Atomic displacement parameters (Å^2^)

**Table:**

	*U*^11^	*U*^22^	*U*^33^	*U*^12^	*U*^13^	*U*^23^
O10	0.0275 (8)	0.0241 (7)	0.0140 (7)	0.0008 (6)	0.0003 (6)	−0.0008 (6)
O11	0.0334 (9)	0.0258 (7)	0.0157 (7)	−0.0013 (7)	0.0032 (7)	0.0015 (6)
O12	0.0247 (8)	0.0274 (7)	0.0153 (7)	−0.0058 (6)	−0.0008 (6)	−0.0013 (6)
O13	0.0196 (8)	0.0349 (9)	0.0150 (7)	0.0060 (6)	−0.0035 (6)	−0.0035 (6)
O14	0.0217 (8)	0.0293 (7)	0.0127 (7)	0.0025 (6)	0.0002 (6)	0.0004 (6)
O15	0.0310 (9)	0.0638 (12)	0.0197 (8)	0.0206 (9)	0.0083 (8)	0.0092 (8)
C5	0.0153 (9)	0.0247 (9)	0.0132 (9)	0.0035 (7)	0.0017 (7)	−0.0002 (8)
C6	0.0175 (10)	0.0236 (9)	0.0133 (9)	0.0003 (8)	−0.0001 (7)	−0.0009 (7)
C7	0.0171 (10)	0.0232 (9)	0.0151 (10)	0.0000 (8)	−0.0009 (8)	−0.0001 (8)
C8	0.0199 (10)	0.0215 (9)	0.0153 (9)	−0.0010 (8)	0.0015 (8)	0.0008 (8)
O16	0.0232 (8)	0.0309 (8)	0.0178 (8)	−0.0013 (7)	0.0002 (7)	−0.0037 (7)
C1	0.026 (2)	0.037 (2)	0.039 (3)	0.0030 (17)	−0.009 (2)	0.004 (3)
C2	0.0195 (19)	0.023 (3)	0.033 (3)	0.0017 (17)	0.000 (2)	0.000 (2)
C3	0.0249 (18)	0.029 (2)	0.044 (2)	0.0039 (15)	0.0006 (17)	−0.0014 (18)
C4	0.031 (2)	0.041 (2)	0.044 (2)	−0.0027 (17)	0.0089 (18)	−0.0054 (19)
N9	0.015 (3)	0.021 (4)	0.017 (2)	−0.0038 (19)	−0.0024 (18)	−0.001 (2)
C1A	0.050 (7)	0.030 (5)	0.040 (7)	0.001 (4)	−0.007 (8)	0.002 (6)
C2A	0.043 (6)	0.033 (7)	0.025 (6)	0.005 (5)	0.001 (5)	0.010 (4)
C3A	0.030 (4)	0.038 (5)	0.030 (5)	0.007 (4)	0.010 (4)	0.000 (4)
C4A	0.065 (8)	0.048 (6)	0.063 (7)	0.027 (5)	0.029 (6)	0.015 (5)
N9A	0.024 (6)	0.021 (9)	0.021 (5)	0.010 (4)	0.003 (4)	−0.003 (5)

### Geometric parameters (Å, º)

**Table:**

O10—C5	1.276 (3)	C3—H3B	0.9900
O11—C5	1.238 (3)	C3—C4	1.513 (6)
O12—H12	0.90 (3)	C4—H4A	0.9800
O12—C6	1.419 (2)	C4—H4B	0.9800
O13—H13	0.85 (3)	C4—H4C	0.9800
O13—C7	1.403 (3)	N9—H9C	0.92 (2)
O14—H14	0.93 (4)	N9—H9A	0.93 (2)
O14—C8	1.299 (3)	N9—H9B	0.91 (2)
O15—C8	1.216 (3)	C1A—H1AA	0.9800
C5—C6	1.530 (3)	C1A—H1AB	0.9800
C6—H6	1.0000	C1A—H1AC	0.9800
C6—C7	1.532 (3)	C1A—C2A	1.517 (14)
C7—H7	1.0000	C2A—H2A	1.0000
C7—C8	1.535 (3)	C2A—C3A	1.518 (12)
O16—H16A	0.87 (4)	C2A—N9A	1.497 (15)
O16—H16B	0.83 (4)	C3A—H3AA	0.9900
C1—H1A	0.9800	C3A—H3AB	0.9900
C1—H1B	0.9800	C3A—C4A	1.514 (12)
C1—H1C	0.9800	C4A—H4AA	0.9800
C1—C2	1.542 (7)	C4A—H4AB	0.9800
C2—H2	1.0000	C4A—H4AC	0.9800
C2—C3	1.513 (6)	N9A—H9AA	0.91 (3)
C2—N9	1.498 (8)	N9A—H9AB	0.90 (3)
C3—H3A	0.9900	N9A—H9AC	0.91 (3)
			
C6—O12—H12	105 (2)	C3—C4—H4B	109.5
C7—O13—H13	112 (2)	C3—C4—H4C	109.5
C8—O14—H14	110 (3)	H4A—C4—H4B	109.5
O10—C5—C6	116.04 (18)	H4A—C4—H4C	109.5
O11—C5—O10	126.33 (19)	H4B—C4—H4C	109.5
O11—C5—C6	117.59 (18)	C2—N9—H9C	116 (5)
O12—C6—C5	110.11 (17)	C2—N9—H9A	108 (3)
O12—C6—H6	108.5	C2—N9—H9B	106 (3)
O12—C6—C7	110.12 (16)	H9C—N9—H9A	108 (3)
C5—C6—H6	108.5	H9C—N9—H9B	111 (4)
C5—C6—C7	110.96 (17)	H9A—N9—H9B	107 (3)
C7—C6—H6	108.5	H1AA—C1A—H1AB	109.5
O13—C7—C6	111.94 (17)	H1AA—C1A—H1AC	109.5
O13—C7—H7	108.8	H1AB—C1A—H1AC	109.5
O13—C7—C8	107.33 (16)	C2A—C1A—H1AA	109.5
C6—C7—H7	108.8	C2A—C1A—H1AB	109.5
C6—C7—C8	111.13 (17)	C2A—C1A—H1AC	109.5
C8—C7—H7	108.8	C1A—C2A—H2A	107.1
O14—C8—C7	114.02 (18)	C1A—C2A—C3A	115.6 (11)
O15—C8—O14	125.2 (2)	C3A—C2A—H2A	107.1
O15—C8—C7	120.83 (19)	N9A—C2A—C1A	109.4 (13)
H16A—O16—H16B	109 (3)	N9A—C2A—H2A	107.1
H1A—C1—H1B	109.5	N9A—C2A—C3A	110.0 (12)
H1A—C1—H1C	109.5	C2A—C3A—H3AA	108.5
H1B—C1—H1C	109.5	C2A—C3A—H3AB	108.5
C2—C1—H1A	109.5	H3AA—C3A—H3AB	107.5
C2—C1—H1B	109.5	C4A—C3A—C2A	115.2 (8)
C2—C1—H1C	109.5	C4A—C3A—H3AA	108.5
C1—C2—H2	108.4	C4A—C3A—H3AB	108.5
C3—C2—C1	112.1 (4)	C3A—C4A—H4AA	109.5
C3—C2—H2	108.4	C3A—C4A—H4AB	109.5
N9—C2—C1	108.4 (5)	C3A—C4A—H4AC	109.5
N9—C2—H2	108.4	H4AA—C4A—H4AB	109.5
N9—C2—C3	111.0 (5)	H4AA—C4A—H4AC	109.5
C2—C3—H3A	108.6	H4AB—C4A—H4AC	109.5
C2—C3—H3B	108.6	C2A—N9A—H9AA	119 (8)
H3A—C3—H3B	107.6	C2A—N9A—H9AB	98 (8)
C4—C3—C2	114.7 (4)	C2A—N9A—H9AC	104 (6)
C4—C3—H3A	108.6	H9AA—N9A—H9AB	111 (4)
C4—C3—H3B	108.6	H9AA—N9A—H9AC	111 (4)
C3—C4—H4A	109.5	H9AB—N9A—H9AC	112 (4)

### Hydrogen-bond geometry (Å, º)

**Table:**

*D*—H···*A*	*D*—H	H···*A*	*D*···*A*	*D*—H···*A*
O12—H12···O11	0.90 (3)	2.00 (3)	2.602 (2)	123 (3)
O13—H13···O16	0.85 (3)	1.83 (3)	2.662 (2)	167 (3)
O14—H14···O10^i^	0.93 (4)	1.58 (5)	2.499 (2)	171 (5)
O16—H16*A*···O15^ii^	0.87 (4)	1.93 (4)	2.791 (2)	169 (4)
O16—H16*B*···O10^iii^	0.83 (4)	2.01 (3)	2.822 (2)	167 (3)
N9—H9*A*···O11	0.93 (2)	1.89 (2)	2.803 (9)	167 (4)
N9—H9*B*···O12^ii^	0.91 (2)	1.97 (3)	2.869 (11)	169 (4)
N9—H9*C*···O13^iv^	0.92 (2)	2.16 (4)	2.922 (13)	140 (5)
N9—H9*C*···O15^iv^	0.92 (2)	2.20 (4)	3.001 (12)	145 (5)
N9*A*—H9*AA*···O11	0.91 (3)	1.87 (4)	2.76 (2)	164 (8)
N9*A*—H9*AB*···O13^iv^	0.90 (3)	1.96 (6)	2.79 (3)	151 (9)
N9*A*—H9*AB*···O15^iv^	0.90 (3)	2.21 (8)	2.83 (3)	126 (6)
N9*A*—H9*AC*···O12^ii^	0.91 (3)	1.99 (5)	2.81 (3)	150 (7)

Symmetry codes: (i) *x*, *y*, *z*−1; (ii) *x*, *y*, *z*+1; (iii) −*x*+2, −*y*+1, *z*; (iv) *x*−1/2, −*y*+1/2, −*z*+1.

## References

[bb1] Ault, A. (1965). *J. Chem. Educ.* **42**, 269.

[bb2] Berkeš, D., Lopuch, J., Proksa, B. & Považanec, F. (2003). *Chem. Pap.* **57**, 350–354.

[bb3] Bruker (2008). *APEX2*, *SAINT* and *SADABS*. Bruker AXS Inc., Madison, Wisconsin, USA.

[bb4] Callear, S. K., Hursthouse, M. B. & Threllfall, T. L. (2008*a*). *University of Southampton, Crystal Structure Report Archive*, 577.

[bb5] Callear, S. K., Hursthouse, M. B. & Threllfall, T. L. (2008*b*). *University of Southampton, Crystal Structure Report Archive*, 582.

[bb6] Campiani, G., Butini, S., Gemma, S., Nacci, V., Fattorusso, C., Catalanotti, B., Giorgi, G., Cagnotto, A., Goegan, M., Mennini, T., Minetti, P., Di Cesare, M. A., Mastroianni, D., Scafetta, N., Galletti, B., Stasi, M. A., Castorina, M., Pacifici, L., Ghirardi, O., Tinti, O. & Carminati, P. (2002). *J. Med. Chem.* **45**, 344–359. 10.1021/jm010982y 11784139

[bb7] Crystal Impact (2014). *DIAMOND*. Crystal Impact, Bonn, Germany.

[bb8] Dolomanov, O. V., Bourhis, L. J., Gildea, R. J., Howard, J. A. K. & Puschmann, H. (2009). *J. Appl. Cryst.* **42**, 339–341.

[bb9] Fogassy, E., Nógrádi, M., Kozma, D., Egri, G., Pálovics, E. & Kiss, V. (2006). *Org. Biomol. Chem.* **4**, 3011–3030. 10.1039/b603058k 16886066

[bb10] Groom, C. R., Bruno, I. J., Lightfoot, M. P. & Ward, S. C. (2016). *Acta Cryst.* B**72**, 171–179. 10.1107/S2052520616003954 PMC4822653 27048719

[bb11] Gül, N. & Nelson, J. H. (1999). *J. Mol. Struct.* **475**, 121–130.

[bb12] Helmkamp, G. K. & Johnson, H. W. Jr (1983). *Selected Experiments in Organic Chemistry*, 3rd ed. New York: W. H. Freeman and Company.

[bb13] Kmecz, I., Simándi, B., Székely, E. & Fogassy, E. (2004). *Tetrahedron Asymmetry*, **15**, 1841–1845.

[bb14] Kokila, L., Cai, S. & Chen, K. (2002). *Chin. J. Chem. Eng.* **10**, 244–248.

[bb15] Mei, L., Jie, S. & Ying, J. (2010). *Res. Chem. Intermed.* **36**, 227–236.

[bb16] Molins, E., Miravitlles, C., López-Calahorra, F., Castells, J. & Raventós, J. (1989). *Acta Cryst.* C**45**, 104–106. 10.1107/s0108270188009710 2610953

[bb17] Periasamy, M., Anwar, S. & Reddy, M. N. (2009). *Indian J. Chem. Sect. B*, **48**, 1261–1273.

[bb18] Sheldrick, G. M. (2015*a*). *Acta Cryst.* A**71**, 3–8.

[bb19] Sheldrick, G. M. (2015*b*). *Acta Cryst.* C**71**, 3–8.

[bb20] Turkington, D. E., MacLean, E. J., Lough, A. J., Ferguson, G. & Glidewell, C. (2005). *Acta Cryst.* B**61**, 103–114. 10.1107/S0108768104029684 15659863

[bb21] Yatcherla, S. R., Islam, A., Nageshwar, D. & Hari Babu, B. (2015). *Heteroletters*, **5**, 241–244.

